# Trauma by Couch: A Case Report of a Massive Traumatic Retroperitoneal Hematoma

**DOI:** 10.21980/J84D2Q

**Published:** 2023-07-31

**Authors:** Cassandra Smith, Graham Stephenson, Alisa Wray, Matthew Hatter

**Affiliations:** * University of California, Irvine, Department of Emergency Medicine, Orange, CA

## Abstract

The authors present the case of a 42-year-old male who was evaluated in a community hospital emergency department (ED) with right upper quadrant and flank pain after falling onto his couch. His evaluation included computed tomography (CT) of his abdomen with intravenous contrast that identified a large right retroperitoneal hematoma measuring an impressive 17 centimeters (cm) in length. The patient was transferred to a receiving trauma center. Upon arrival a focused assessment with sonography in trauma (FAST) ultrasound was obtained. The interpretation of the findings was complicated by distortion of his anatomy by the hematoma. The patient remained hemodynamically stable and was admitted for continued observation. He was ultimately discharged home in stable condition. This case report provides a concise overview of the approach to evaluating blunt abdominal trauma, imaging considerations, and a brief review of the management of retroperitoneal hematomas.

**Topics:**

Trauma, retroperitoneal hemorrhage, ultrasound, FAST, computed tomography, hepatorenal recess, Morrison’s pouch.

[Fig f1-jetem-8-3-v1] [Fig f2-jetem-8-3-v1]

**Figure f1-jetem-8-3-v1:**
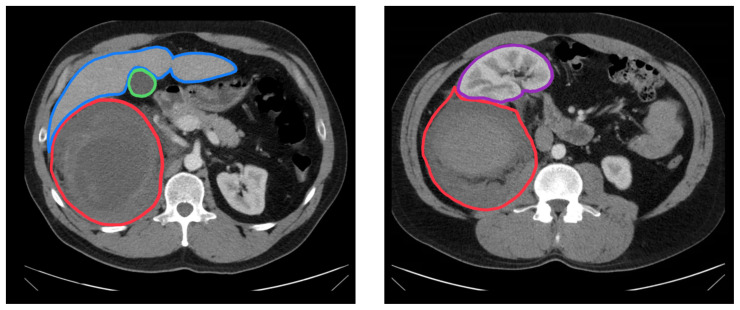
Unannotated CT Video Link: https://youtu.be/fKWMmcR8T4M Annotated CT Video Link: https://youtu.be/bZGjvnpo1B0

**Figure f2-jetem-8-3-v1:**
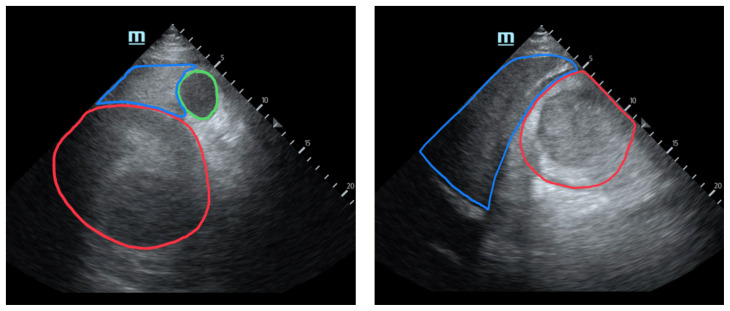
Unannotated Ultrasound 1 Video Link: https://youtu.be/PpEbnE6BDUo Annotated Ultrasound 1 Video Link: https://youtu.be/vj4mDGYWUH4 Unannotated Ultrasound 2 Video Link: https://youtu.be/mWY5-pl_9Do Annotated Ultrasound 2 Video Link: https://youtu.be/IWypm1ckbyg

## Brief introduction

The retroperitoneal space is defined posteriorly by the transversalis fascia covering the posterior abdominal wall, and anteriorly by the parietal peritoneum.^1^ This space contains the kidneys, ureters, suprarenal glands and many neurovascular structures such as the aorta and lumbar plexus.^1^,^2^ The retroperitoneal space is further subdivided into five compartments — the lateral three of which are divided by renal fascia (anterior pararenal space, perirenal space, and posterior pararenal space), the great vessel compartment from T12-L4/5 between perirenal spaces, and posterior compartment (behind transversalis fascia) containing the psoas major which joins the iliacus distally. 3,4,5

Traumatic retroperitoneal injuries are further delineated into “zones.” Zone 1 is comprised of the central space between the diaphragm to the bifurcation of the aorta. Zone 2 is not contiguous and contains the lateral segments of the retroperitoneum including the perinephric areas, kidneys, renal vessels, and portions of the colon. Zone 3 is inferior to the aortic bifurcation extending to include the right and left internal and external iliac vasculature, sigmoid colon, and rectum.^6^

In blunt abdominal trauma, organ injury or disruption of the vasculature will result in bleeding and hematoma formation. Typically, blunt injuries will remain contained in a single zone. This is in contrast to penetrating injuries that often cross into neighboring zones. 6

Diagnosis of retroperitoneal injury is typically through advanced imaging with computed tomography (CT). This modality confers the benefit of rapid image acquisition and provides information on vessels and viscus involved in the injury. Intravenous contrast may further assist the provider by the presence or absence of active extravasation, signaling if active bleeding is ongoing.^1^,^3^,^5^,^6^

Management of bleeding into the retroperitoneal space may be managed operatively, endovascularly, or simply observed. The zone involved, hemodynamic stability of the patient, and evidence of active extravasation into the hematoma are all considered in management.^6^

## Presenting concerns and clinical findings

This case describes a 42-year-old male with no past medical history who presented to the emergency department (ED) after blunt trauma. Earlier in the evening, he slipped on his couch and landed awkwardly, striking his right flank on the hard frame of the structure. He experienced immediate discomfort to his right flank which worsened over time. He was taken to a community emergency department (non-trauma center). Documentation from this visit included a physical exam describing right flank tenderness to palpation and mild distention of his right upper quadrant (RUQ). His workup included labs, a computed tomography (CT) scan with intravenous (IV) contrast of his abdomen, and radiographs of his right elbow. Complete blood count (CBC) revealed a hemoglobin of 13.5 g/dL and hematocrit of 39%. Comprehensive metabolic panel and coagulation studies were within normal limits. Radiographs of his right elbow did not show evidence of an acute fracture or dislocation. Computed tomography of the abdomen and pelvis with IV contrast revealed a large right retroperitoneal hematoma, approximately 17 cm in diameter, without blush of contrast. After identification of the retroperitoneal hematoma, he was transferred to a trauma center for further evaluation and management.

## Significant findings

Upon arrival at the trauma center, a FAST revealed a large, well-circumscribed abnormality (red outline) deep to the liver (blue outline and star) and gallbladder (green outline and star). The right kidney and hepatorenal space were not clearly visualized. The remainder of the FAST showed no free fluid in the splenorenal space, pelvis, and no pericardial effusion. He had lung sliding bilaterally.

Computed tomography with IV contrast of the abdomen and pelvis was repeated, confirming the right-sided retroperitoneal hematoma (red outline). Specifically, it was contained in the perirenal space in direct contact with the liver and the prerenal space (convergence of the red outline and blue outline). At 17cm in diameter, the hematoma was sufficiently large that it displaced the right kidney anteriorly and towards the anterior midsternal line (purple outline and star) into an area normally occupied by the small intestine. Again, there was no active extravasation of contrast noted on the CT. The gallbladder was without abnormal findings (green outline). No liver lacerations or hematomas were noted (blue outline and star). There were no obvious rib fractures.

## Patient course

The patient was admitted to the trauma service where he underwent serial abdominal exams and hemograms. His abdomen remained soft and minimally tender. His hemoglobin and hematocrit remained stable at 13 g/dL and 38%, respectively, for three consecutive lab draws taken at six-hour intervals. During his observation, he was ambulatory and tolerating food and fluids. He was discharged after 23 hours and advised to follow up in the trauma clinic. He was seen one week later at which time he reported minimal pain and was able to perform his activities of daily living. His hemoglobin at that time was 13.1 g/dL.

## Discussion

The evaluation of blunt trauma per Advanced Trauma Life Support (ATLS) includes a primary survey, secondary survey, and adjunct imaging, most commonly including ultrasound (FAST) and CT. It is estimated that up to 13% of patients with blunt abdominal trauma will have some form of intra-abdominal injury, with 4.7% requiring intervention for management.^7^ Physical exam findings that may direct the physician toward retroperitoneal injury include instability of the pelvis, Cullen’s sign (discoloration around navel), Grey-Turner’s sign (hematoma around lateral abdominal wall), or Bryant’s sign (scrotal fullness and ecchymosis). 8,9 These findings are often late presentations of associated retroperitoneal bleeding and inconsistent markers for injury.

Ultrasound (US) is a valuable tool in the evaluation of blunt abdominal trauma, but its effectiveness in assessing retroperitoneal injuries is limited due to a variety of factors. These include provider experience levels, patient positioning and body type, limited acoustic windows for evaluation of the retroperitoneum, and hematoma volumes.^10^–^12^ Furthermore, US is limited in its ability to detect active bleeding at a site of injury. Studies have shown that roughly one-third of patients with retroperitoneal injuries, including injuries of the duodenum and pancreas, will have normal FAST examinations.^13^,^14^ Due to these limitations, computed tomography is considered the standard of care in the evaluation of retroperitoneal injuries. The speed of acquisition, high spatial resolution, and noninvasive nature of the study contribute to its utility in directing management and identifying injuries.^11^,^15^–^18^

In general, the sonographic appearance of a hematoma will vary depending on the age of the injury. Initially, injuries will exhibit mixed echogenicity and, over time, will become more hypoechoic as the clot matures. Certain sonographic signs are associated with hematomas such as the “hematocrit sign” (layering of cellular debris) and “plankton sign” (mobile proteinaceous debris within the hematoma).^19^

Retroperitoneal injuries resulting from blunt trauma have a high degree of morbidity and mortality.^20^ Management decisions are made on a variety of factors, including hemodynamic stability, active contrast extravasation into the injury, and the areas involved in the injury. Retroperitoneal zones, as described above in the introduction, will often direct the provider as to the appropriate approach for management. Management may range from simple observation, to open laparotomy, pelvic packing, angioembolization, or resuscitative endovascular balloon occlusion of the aorta (REBOA). 18

This case is remarkable for a large retroperitoneal hematoma captured both on ultrasound and CT. The patient was hemodynamically stable and ultimately did not require operative or invasive intervention in his recovery.

## Supplementary Information




























