# Supplementary figures and images for: Trauma by Couch: A Case Report of a Massive Traumatic Retroperitoneal Hematoma
Source: J Educ Teach Emerg Med. 2023 Jul 31;8(3):V5–9. doi: 10.21980/J84D2Q (PMC10414980; doi:10.21980/J84D2Q)

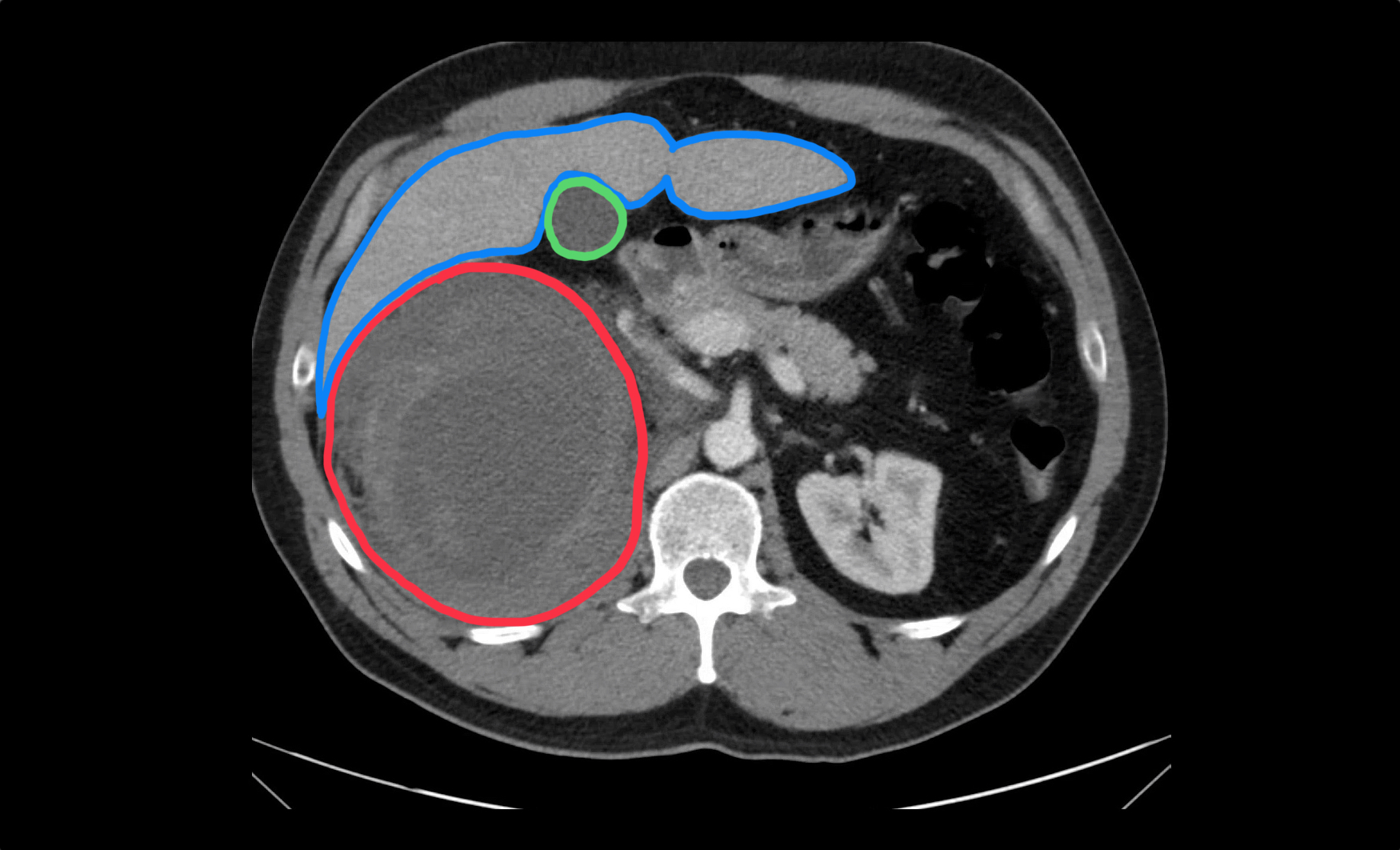

Supplement: Supplementary file 1 [file JETem-8-3-V1-supp1.jpeg]

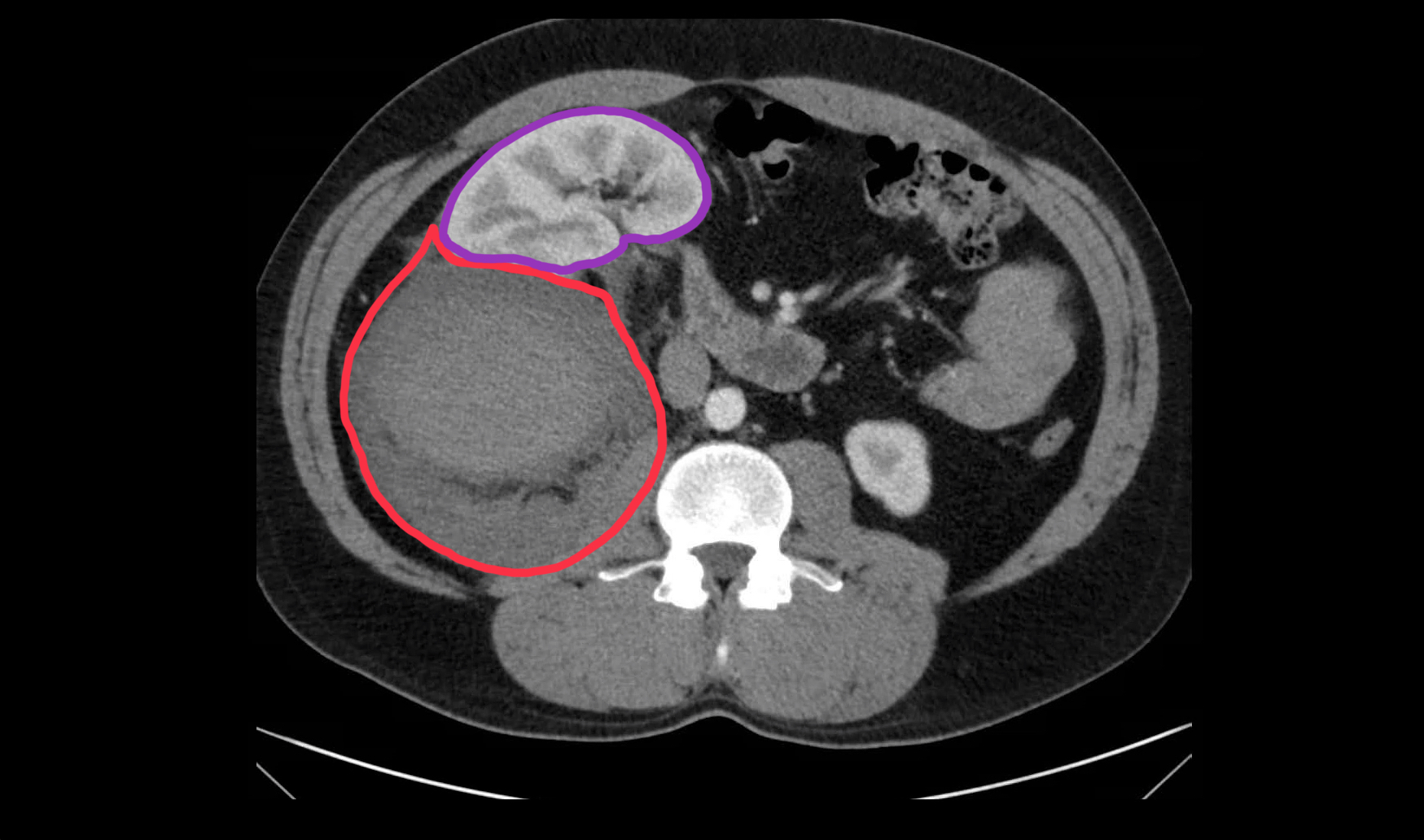

Supplement: Supplementary file 2 [file JETem-8-3-V1-supp2.jpeg]

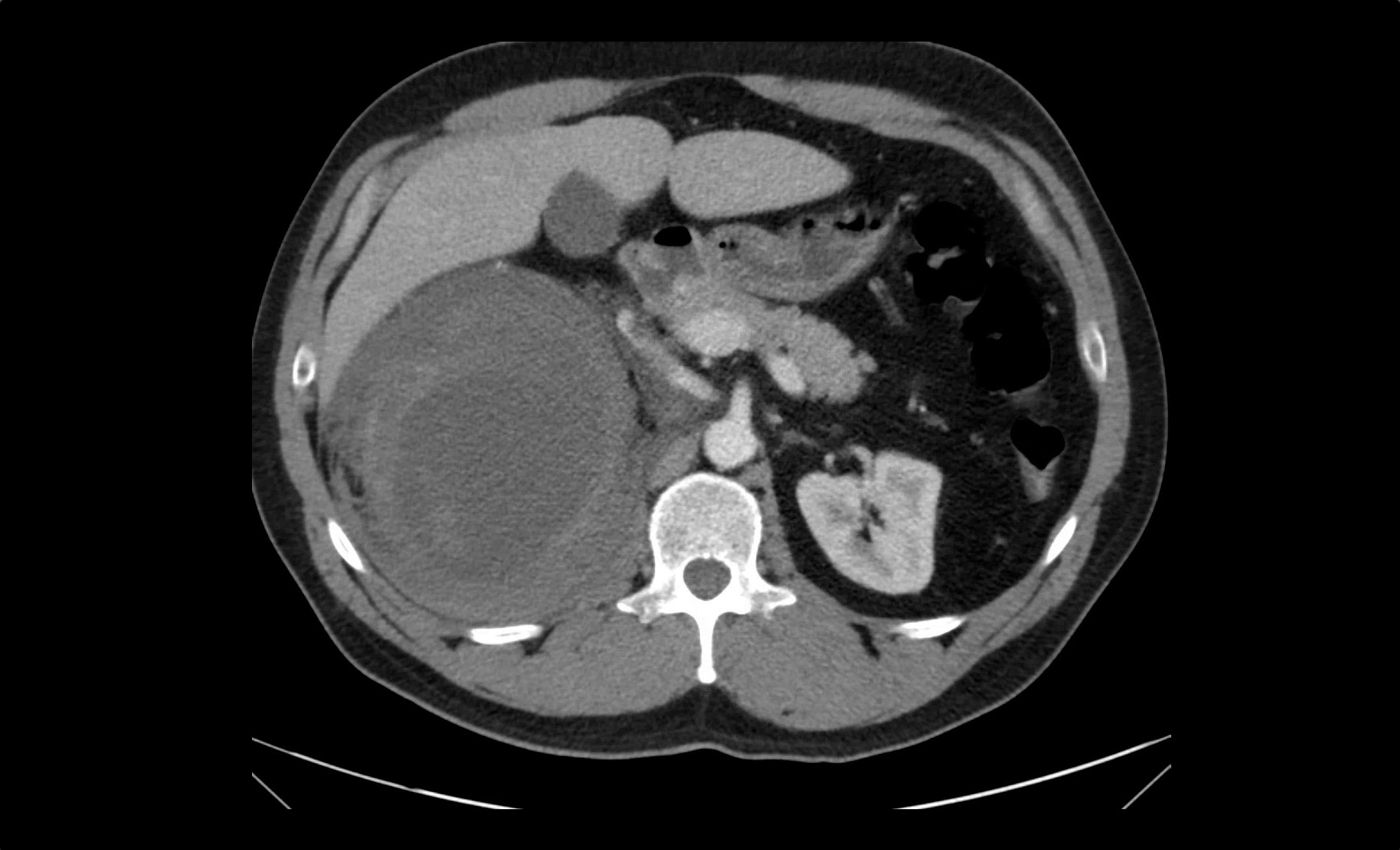

Supplement: Supplementary file 4 [file JETem-8-3-V1-supp4.jpeg]

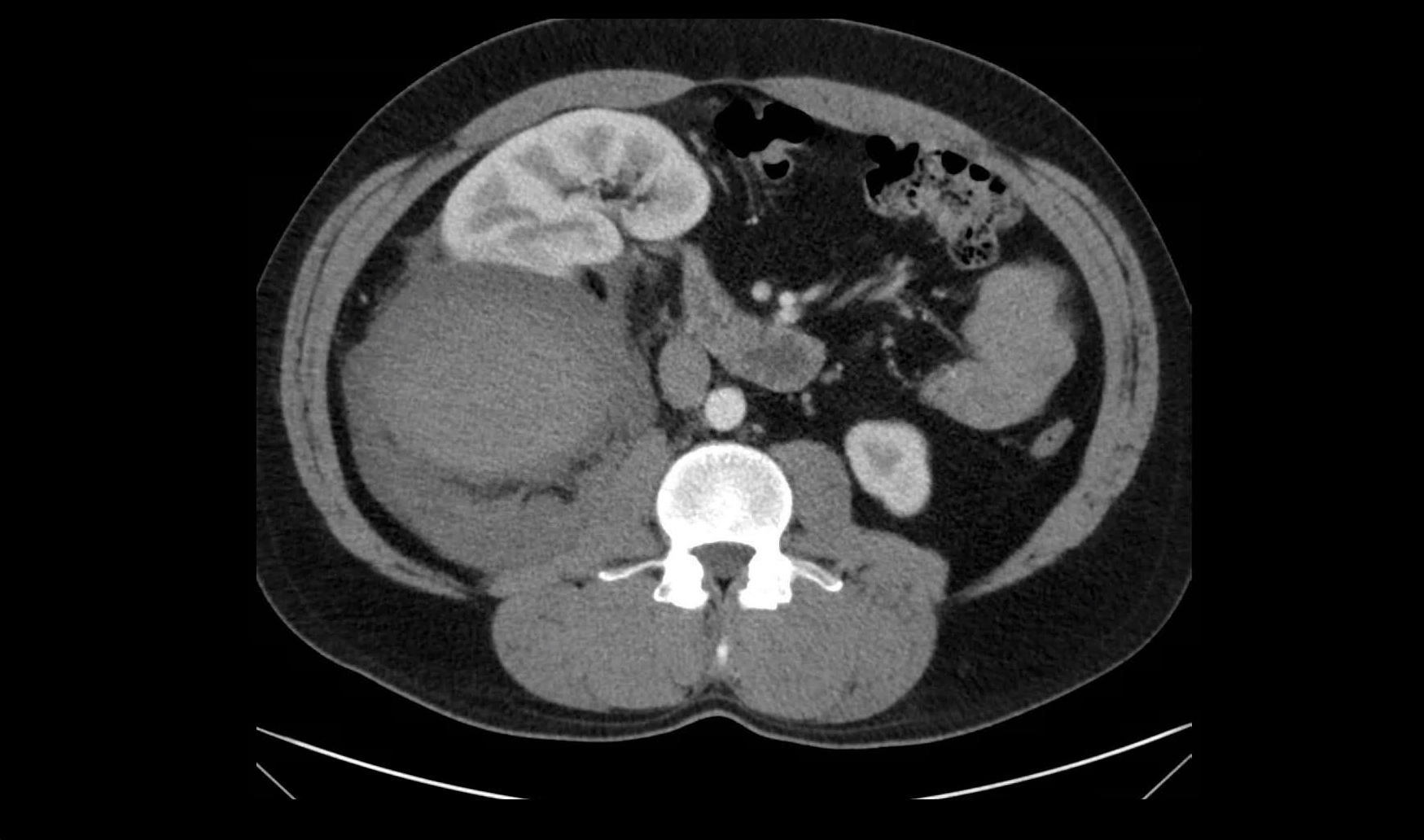

Supplement: Supplementary file 5 [file JETem-8-3-V1-supp5.jpeg]

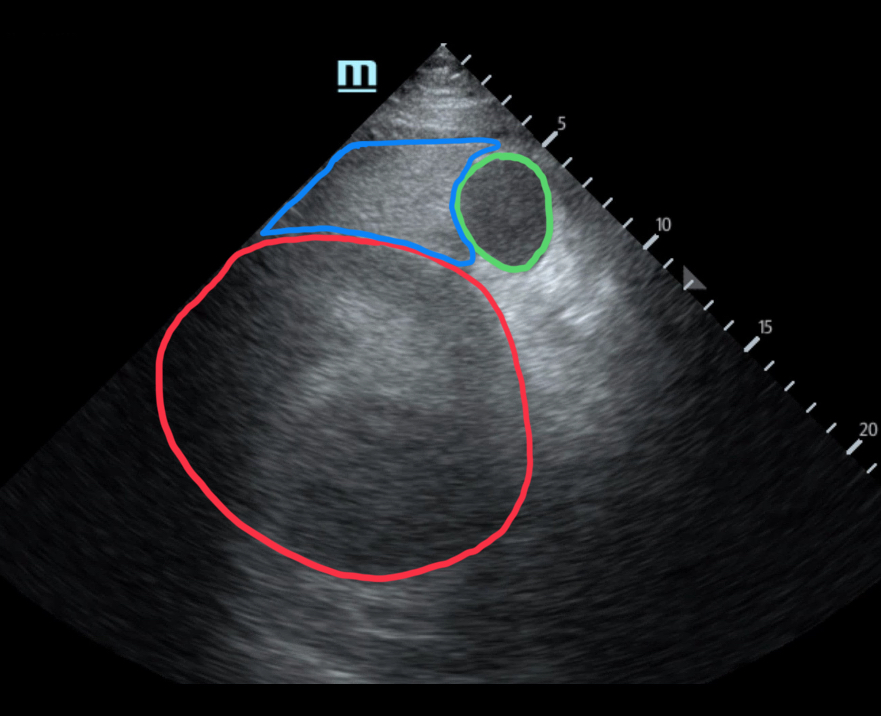

Supplement: Supplementary file 7 [file JETem-8-3-V1-supp7.jpeg]

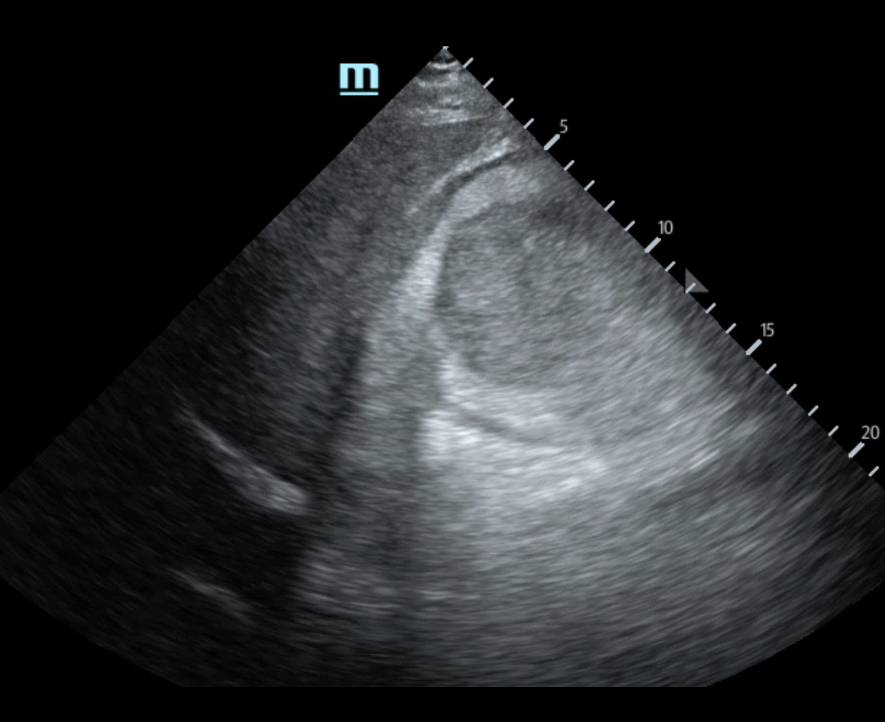

Supplement: Supplementary file 9 [file JETem-8-3-V1-supp9.jpeg]

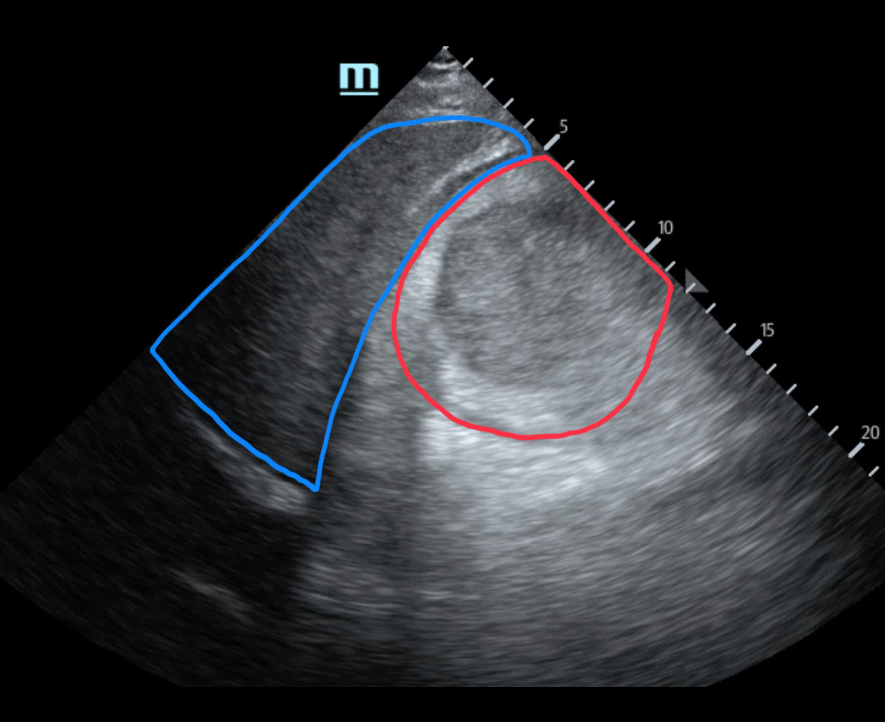

Supplement: Supplementary file 11 [file JETem-8-3-V1-supp11.jpeg]

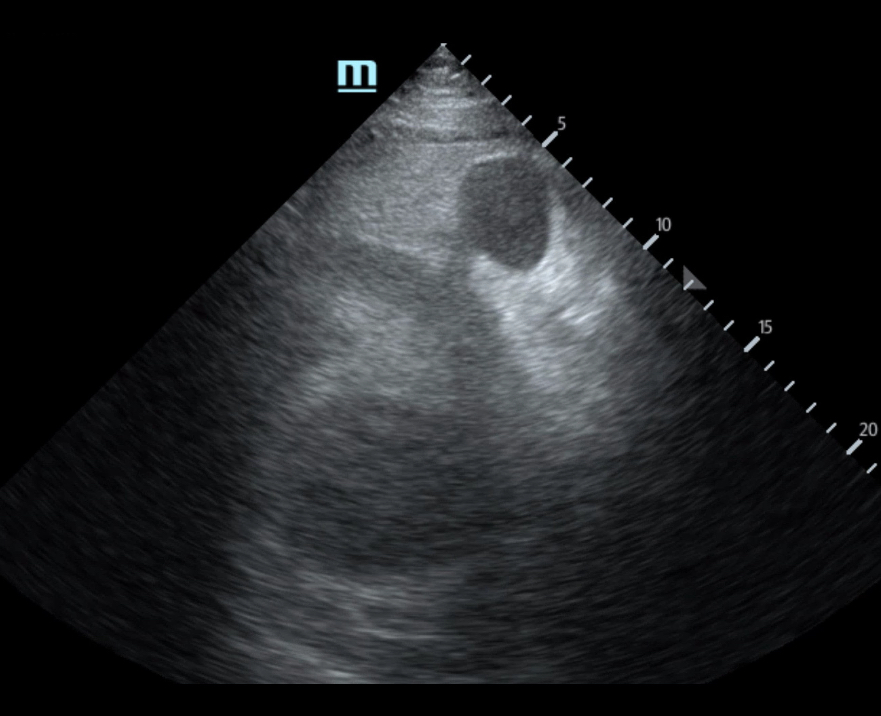

Supplement: Supplementary file 13 [file JETem-8-3-V1-supp13.jpeg]
